# Identification of FPR3 as a Unique Biomarker for Targeted Therapy in the Immune Microenvironment of Breast Cancer

**DOI:** 10.3389/fphar.2020.593247

**Published:** 2021-02-11

**Authors:** Jian Qi, Yu Liu, Jiliang Hu, Li Lu, Zhen Dou, Haiming Dai, Hongzhi Wang, Wulin Yang

**Affiliations:** ^1^ Anhui Province Key Laboratory of Medical Physics and Technology, Institute of Health and Medical Technology, Hefei Institutes of Physical Science, Chinese Academy of Sciences, Hefei, China; ^2^ Scinece Island Branch, Graduate School of USTC, Hefei, China; ^3^ Hefei Cancer Hospital, Chinese Academy of Sciences, Hefei, China; ^4^ Department of Neurosurgery, The Shenzhen People’s Hospital (The Second Clinical Medical College of Jinan University), Shenzhen, China; ^5^ Department of Anatomy, Shanxi Medical University, Taiyuan, China; ^6^ Hefei National Science Center for Physical Sciences at the Microscale, University of Science and Technology of China, Hefei, China

**Keywords:** breast cancer, immune microenvironment, ESTIMATE algorithm, immune checkpoint, FPR3

## Abstract

Although research into immunotherapy is growing, its use in the treatment of breast cancer remains limited. Thus, identification and evaluation of prognostic biomarkers of tissue microenvironments will reveal new immune-based therapeutic strategies for breast cancer. Using an in silico bioinformatic approach, we investigated the tumor microenvironmental and genetic factors related to breast cancer. We calculated the Immune score, Stromal score, Estimate score, Tumor purity, TMB (Tumor mutation burden), and MATH (Mutant-allele tumor heterogeneity) of Breast cancer patients from the Cancer Genome Atlas (TCGA) using the ESTIMATE algorithm and Maftools. Significant correlations between Immune/Stromal scores with breast cancer subtypes and tumor stages were established. Importantly, we found that the Immune score, but not the Stromal score, was significantly related to the patient's prognosis. Weighted correlation network analysis (WGCNA) identified a pattern of gene function associated with Immune score, and that almost all of these genes (388 genes) are significantly upregulated in the higher Immune score group. Protein-protein interaction (PPI) network analysis revealed the enrichment of immune checkpoint genes, predicting a good prognosis for breast cancer. Among all the upregulated genes, FPR3, a G protein-coupled receptor essential for neutrophil activation, is the sole factor that predicts poor prognosis. Gene set enrichment analysis analysis showed FRP3 upregulation synergizes with the activation of many pathways involved in carcinogenesis. In summary, this study identified FPR3 as a key immune-related biomarker predicting a poor prognosis for breast cancer, revealing it as a promising intervention target for immunotherapy.

## Introduction

Breast cancer, the most common gynecological cancer worldwide (Ghoncheh et al., 2016), is becoming a majorpublic health crisis, and the number of new cases diagnosed each year is ever-increasing (Cheng et al., 2018). Breast cancer is considered less immunogenic than melanoma or renal cell carcinoma, and the results of adoptive immunotherapy (interleukin-2, interferon) have been relatively disappointing (Burugu et al., 2017). Over the past decade, with the increased understanding of the immune microenvironment of breast cancer tissues, immune escape has been considered an important feature of breast cancer development (Romaniuk and Lyndin, 2015; Takada et al., 2018). Targeting the tumor immune microenvironment in breast cancer is of high therapeutic interest (Zhao et al., 2017). However, the therapeutic effects of immune checkpoint inhibition may be limited. For example, the evaluation of avelumab, an anti-PDL1 antibody, in various subtypes of breast cancer showed that the overall response rate (ORR) for the entire cohort was 4.8% (Emens, 2018), far from achieving the intended effect.

The tumor microenvironment is composed of a variety of immune cells and stromal cells, endothelial cells along with inflammatory mediators, and extracellular matrix (ECM) molecules (Cooper et al., 2012; Straussman et al., 2012). It plays a key role in altering the tumor response to treatment (Hanahan and Weinberg, 2000; Hanahan and Coussens, 2012). Previous studies have shown that high levels of immune cell infiltration are associated with better prognosis for diseases such as (Tas and Erturk, 2017) prostate cancer (Donovan et al., 2018), cutaneous melanoma (Yang et al., 2020), and breast cancer (Papatestas et al., 1976; Manuel et al., 2012). Also, high immune infiltration is associated with neoadjuvant chemotherapy and an enhanced response to adjuvant chemotherapy (Pruneri et al., 2018). Hence, assessing TME heterogeneity and reshaping the immune microenvironment may hold promise for cancer treatments in the near future. ESTIMATE (Estimate of Stromal and Immune Cells in Malignant Tumor Tissues from Expression Data) is a newly developed algorithm that applies gene expression data to predict the fraction of stromal and immune cells in tumor samples (Yoshihara et al., 2013). Weighted gene co-expression network analysis (WGCNA) is an effective tool to establish correlation patterns between genes to identify cancer-related modules and central genes (Langfelder and Horvath, 2008). Combining multiple bioinformatic approaches, we explored the microenvironment and genetic factors associated with breast cancer to determine the prognostic biomarkers for breast cancer.

## Materials and Methods

### Gene Expression Datasets

The level 3 gene expression profile (level 3 data) for breast cancer was obtained from the UCSC Xena (https://xenabrowser.net/datapages/). Clinical data, such as gender, race, age, histological type, survival, and outcome, were downloaded from the TCGA data portal. Count data were used to quantitate a total of 19,986 protein-coding genes that had been annotated in the Ensembl database (http://asia.ensembl.org/index.html). The ESTIMATE algorithm was applied to the normalized expression matrix to determine the Immune/Stromal scores for each breast cancer sample. Immune score and Stromal score were calculated by applying the ESTIMATE algorithm to the downloaded database (Yoshihara et al., 2013). To verify the association between FPR3 mRNA expression and survival, a dataset, GSE11121 (contains 200 samples), was obtained from the Gene Expression Omnibus (GEO) database for testing.

### Correlations Between Prognosis and Immune/Stromal Score

The overall survival rate was taken as the main prognostic indicator. We divided the relevant patients into two groups based on the Immune/Stromal scores of each breast cancer sample. Kaplan-Meier plots were generated to illustrate the relationship between patients’ overall survival and gene expression levels of differentially expressed genes (DEGs). The relationship was tested by the log-rank test.

### Identification of Differentially Expressed Genes (DEGs)

According to the ESTIMATE results, all samples were divided into high/low immune-score groups and high/low stromal-score groups to select the intersection genes. *p* Value <0.05, Fold change >2 were set as the cutoffs to identify significantly differentially expressed genes. Heatmaps were generated using the pheatmap package in R software (Galili et al., 2018) (cran.r-project.org/web/package/pheatmap/index.html), and the limma package (Ritchie et al., 2015) (limma package; www.r-project) was used to separate the upregulated and downregulated genes in the high score. Venn diagrams were drawn up with web tools (http://bioinformatics.psb.ugent.be/webtools/Venn/). The WGCNA method (Langfelder and Horvath, 2008) was used to construct the co-expression network of the genes in the test samples of breast cancer.

### Gene Ontology and KEGG Pathway Enrichment Analysis

In our study, Gene Ontology (GO) analysis and the Kyoto Encyclopedia of Genes and Genomes (KEGG) analysis were employed to understand the potential function of genes using the clusterProfiler package in R software (Yu et al., 2012). Moreover, to analyze the interaction genes, the protein-protein interaction (PPI) network was built using STRING (Szklarczyk et al., 2019) and reconstructed in Cytoscape v3.6, as previously stated (Shannon et al., 2003; Liu et al., 2020). Finally, Molecular Complex Detection (MCODE) in Cytoscape was utilized to obtain clusters based on the topology to localize densely connected regions.

### Gene Expression Analysis in GEPIA

The online database Gene Expression Profiling Interactive Analysis (GEPIA) (http://gepia.cancer-pku.cn/index.html) (Tang et al., 2017) is an interactive website that includes 9,736 tumors and 8,587 normal samples from the TCGA portal and the GTEx project. GEPIA is based on gene expression with the log-rank test and the MantelCox test in 33 different types of cancer. Gene expression correlation analysis was performed for a given set of TCGA expression data. The Spearman method was used to determine the correlation coefficient.

### Gene Set Enrichment Analysis

GSEA is a calculation method used in determining whether a set of basically defined gene sets exhibit statistically significant differences between two biological states (Subramanian et al., 2005). In this study, GSEA first generated an ordered list of all genes based on their correlation with FPR3 expression. Gene set permutations were performed 1,000 times for each analysis. The expression level of FPR3 is deemed to be a phenotypic marker. The nominal *p* value and normalized enrichment score (NES) served to sort the pathways enriched in each phenotype.

### Statistical Analysis

All data are expressed as mean ± SD (standard deviation). All analyses were performed with R version 3.5.3 (http://www.R-project.org). Immune and Stromal scores were calculated by using the ESTIMATE package. Data were analyzed with standard statistical tests where appropriate. Multiple testing was adjusted by using the FDR method.

## Results

### Immune and Stromal Score Correlate With Breast Cancer Subtypes

**FIGURE 1:**
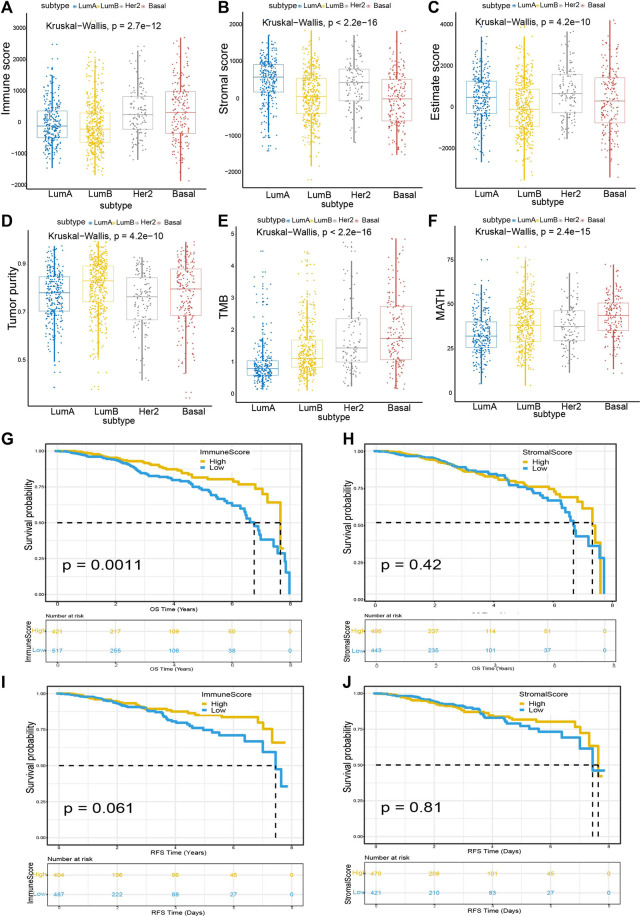
Tumor microenvironmental factors were tightly associated with breast cancer subtypes and Immune score **(A)** Box-plot shows a significant association between breast cancer subtypes and the level of Immune score (n = 1,040, *p* = 2.7*e* − 12) **(B)** Box-plot reveals the significant association between breast cancer subtypes and the level of Stromal score (n = 1,040, *p* < 2.2*e* − 16) **(C–F)** Box-plot shows a significant correlation between breast cancer subtype and Estimate score (n = 1,040, *p* = 4.2*e* − 10), Tumor purity (n = 1,040, *p* = 4.2*e* − 10), TMB (n = 1,040, *p* < 2.2*e* − 16), MATH (n = 1,040, *p* = 2.4*e* − 15) **(G)** Breast cancer cases were divided into two groups based on their average expression of Immune scores. A high Immune score predicts a favorable prognosis for overall survival (OS). As indicated by the log-rank test, *p* = 0.0011 **(H)** Similarly, breast cancer cases were parted into two groups based on their average expression of Stromal scores. The survival time for OS of the high Stromal score group is longer than that of the low Stromal score group, although the difference was not statistically significant (*p* = 0.42) **(I)** A similar grouping based on Immune scores was used for RFS (relapse-free survival) survival analysis **(J)** A similar grouping based on Stromal scores was used for RFS survival analysis.

We found significant correlations between Immune score and Stromal score with breast cancer subtypes. In this study, we obtained information about 1,040 breast cancer patients from the TCGA database, including their gene expression signatures and clinical profiles. The average age of the patients was 58.34 years. Using the PAM50 classification method, we used the genefu package (Gendoo et al., 2016) in R language to divide the breast cancer subtypes into luminal A (LumA), luminal B (LumB), Her2+, and Basal. The number of patients with luminal A was 286, the number of patients with subtype luminal B was 427, the number of patients with Her2+ was 128, and the number of patients with Basal was 199. According to the calculation results of the ESTIMATE and maftools (Mayakonda et al., 2018) algorithm, as shown in Figures 1A–F, the Immune score, Stromal score, Estimate score, Tumor purity, TMB, and MATH are significantly correlated with different breast cancer subtypes. Among the Immune score, the basal subtype had the highest average Immune score, followed by the Her2+ subtype, LumA third, and LumB the lowest (*p* = 2.7*e* − 12); Similarly, in breast cancer subtypes, the order of Stromal score from high to low is LumA > Her2+ > LumB > Basal (*p* < 2.2*e* − 16). In Estimate score, the score from high to low is HER2+ > Basal > LumA > LumB (*p* = 4.2*e* − 10); in Tumor purity, the score from high to low is LumB > Basal > LumA > Her2+ (*p* = 4.2*e* − 10). In TMB, the score from high to low is Basal > Her2+ > LumB > LumA (*p* < 2.2*e* − 16), while in MATH, the score from high to low is Basal > LumB > Her2+ > LumA (*p* = 2.4*e* − 15).

Next, to explore the correlations between Immune/Stromal scores and breast cancer prognosis, we constructed survival curves of patients by classifying them into high and low score groups based on their gene expression profiles. We found that patients with higher Immune scores have longer survival rates for OS (Overall survival) than those with lower Immune scores (*p* < 0.0011). Similarly, patients with high Stromal scores have better life expectancy than patients with low Stromal scores, although the data is not statistically significant (Figures 1G,H). In addition, we also calculated survival curves for RFS (relapse-free survival), DFS (Disease-free survival), and PFS (progression-free survival). We can see that the higher the Immune score, the better the survival rates for RFS, DFS, and PFS. The results are shown in Figures 1I,J (RFS) and supplementary Figure S1 (DFS and PFS). Although the *p* value is not significant, it is close to 0.05.

### The Significant Correlation Between Immune/Stromal Scores and T Stage of Breast Cancer

**FIGURE 2:**
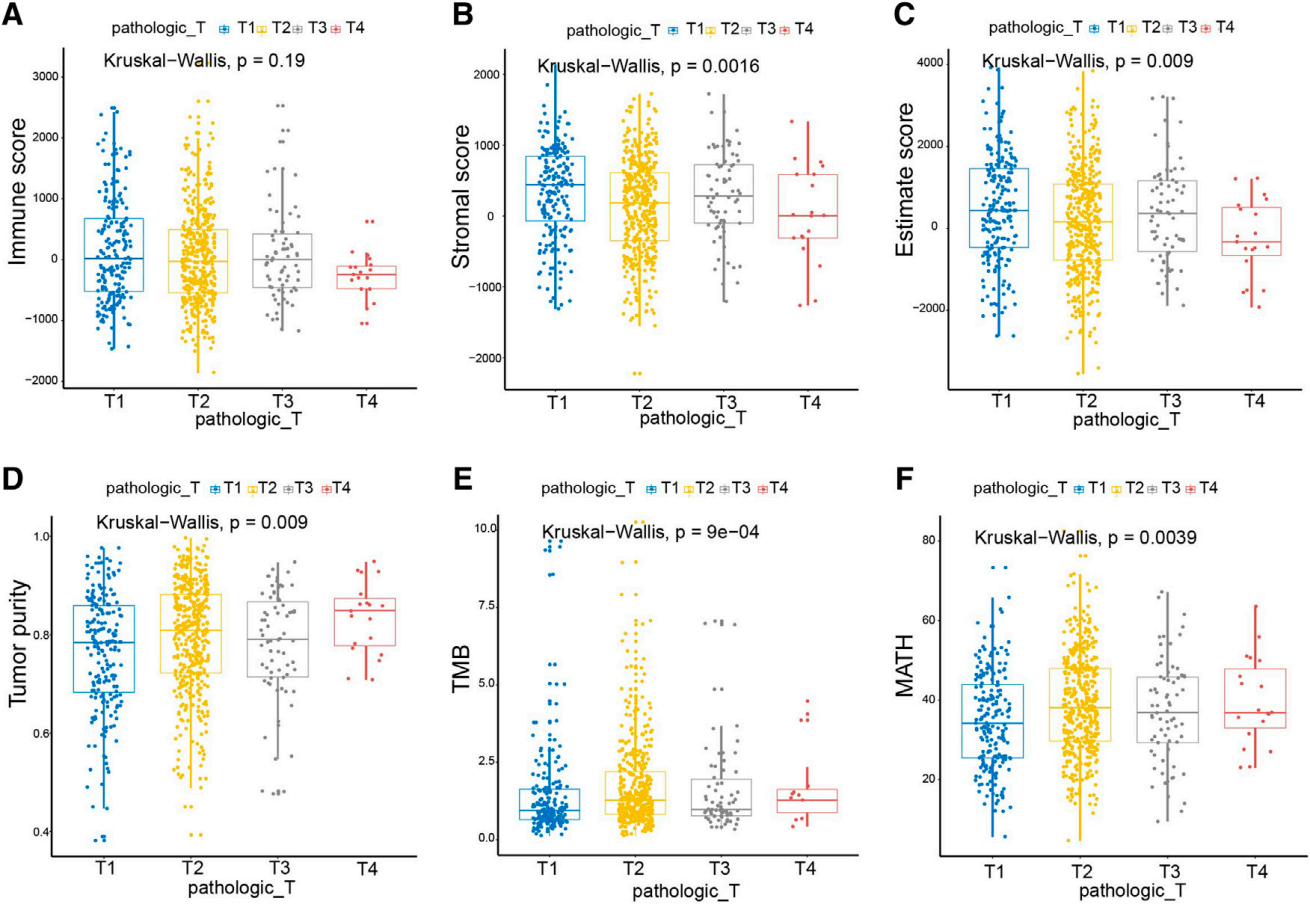
Immune and Stromal scores were significantly associated with the T stages of breast cancer. As the T stage progressed, the Immune score, Stromal score, and Estimate score decreased, and the scores of Tumor purity, TMB, and MATH increased. Pathologic_T, pathological T staging of tumor, represented by T1-T4 in turn. Kruskal_Wallis, the kruskal wallis test was used to compare multi-independent samples.

The TNM staging system is a globally recognized standard for classifying the extent of the spread of cancer into nearby tissue. Given the correlations of both Immune- and Stromal scores with breast cancer subtypes, we studied their connection to TNM stages of breast cancer. We noted that the Immune score, Stromal score, and Estimate score decrease following the T stage progression (Figures 2A–C). In line with our observation in Figure 1G, the higher the Immune score, the better the prognosis. Consistently, the Tumor purity, TMB, and MATH increased with the progression of T stages, correlating well with the degree of malignancy of breast cancer (Figures 2D–F). The M and N stages of the tumor are only correlated with TMB, but not related to the Immune score, Stromal score, Estimate score, tumor purity, or MATH (Supplementary Figure S2).

### Analysis of Differentially Expressed Genes

**FIGURE 3:**
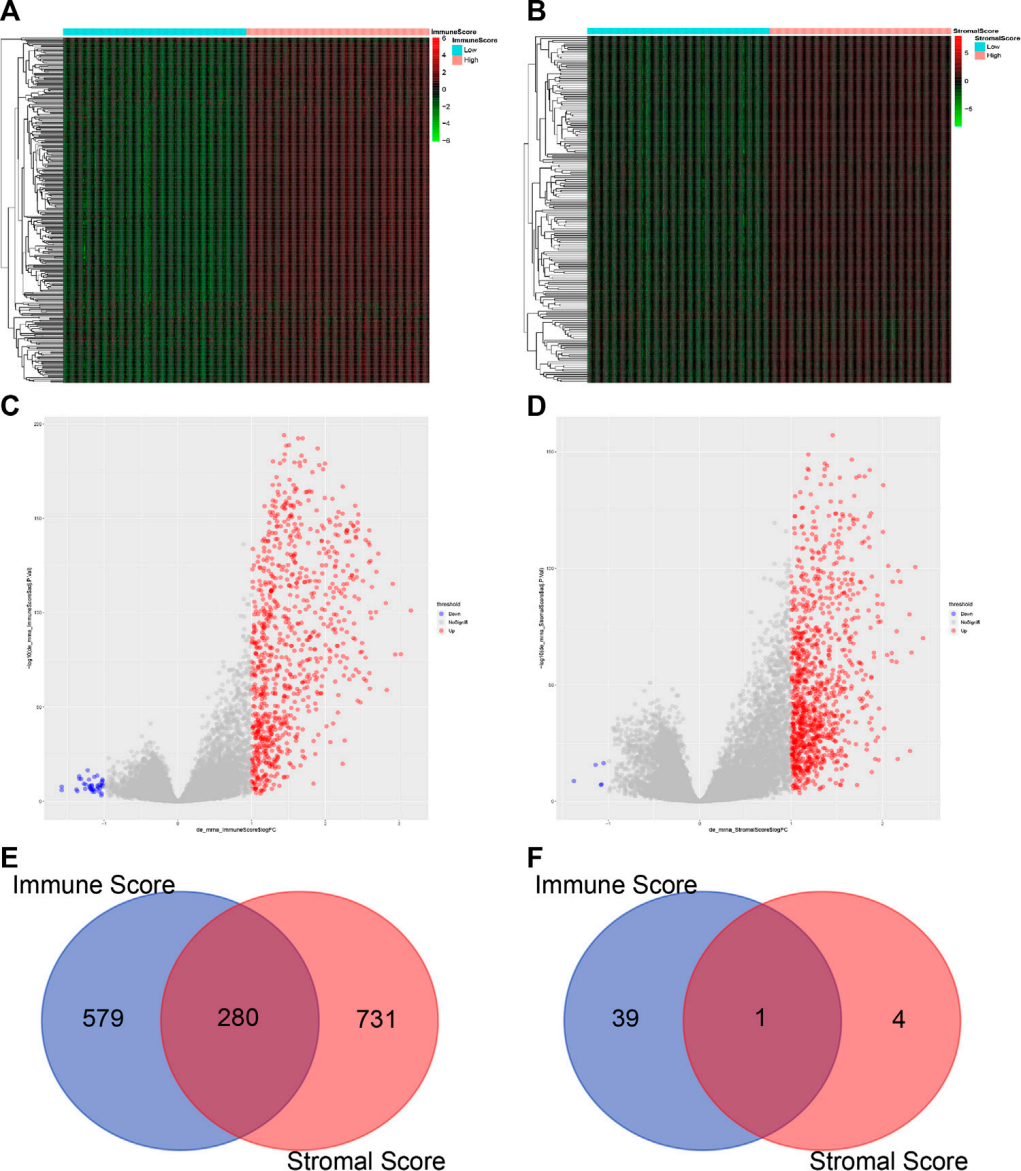
Comparison of gene expression profiles in high vs. low Immune or Stromal scores **(A)** Heatmap of the DEGs in the Immune score of high vs. low. Genes upregulated are displayed in red; genes downregulated are shown in green. Genes with no change are in black (*p* < 0.05, FC > 2). FC, fold change **(B)** Heatmap of the DEGs in Stromal score of high vs. low (*p* < 0.05, FC > 2) **(C–D)** Volcano plot of gene expression profiles in high vs low Immune or Stromal scores. A total of 859 genes were upregulated and 40 genes were downregulated in the group of high Immune score (FC > 2, *p* < 0.05). Similarly, there were 1,011 upregulated genes and five downregulated genes in the high Stromal score group (FC > 2, *p* < 0.05) **(E–F)** Venn diagrams display the number of commonly upregulated **(C)** or downregulated **(D)** DEGs in the high Immune or Stromal score groups.

To determine the correlation between the overall gene expression profile and the Immune/Stromal scores, we evaluated the sequencing data of 1,040 breast cancer patients in the TCGA database. In this analysis, immune-related genes were divided into the high-score and the low-score group. We then used the limma package of R language to analyze the differentially expressed genes. Heatmaps in Figures 3A,B show distinct gene expression profiles of DEGs corresponding to the high vs. low Immune score/Stromal score groups. A total of 859 genes were upregulated and 40 genes were downregulated in the group of high Immune score (FC > 2, *p* value < 0.05). Similarly, there were 1,011 upregulated genes and five downregulated genes in the high Stromal score group (FC > 2, *p* value < 0.05) (Figures 3C,D).

We found that 280 genes were upregulated in the groups with both high Immune score and high Stromal score (Figure 3E) and only one common gene was downregulated (Figure 3F). On the other hand, the Stromal score was not significantly related to the prognosis of breast cancer patients (Figures 1G,H). Therefore we decided to exclude the Stromal score from our analysis when verifying gene modules associated with Immune score.

### Functional Enrichment Analysis Reveals FPR3 as a Key Immune-Related Gene

**FIGURE 4:**
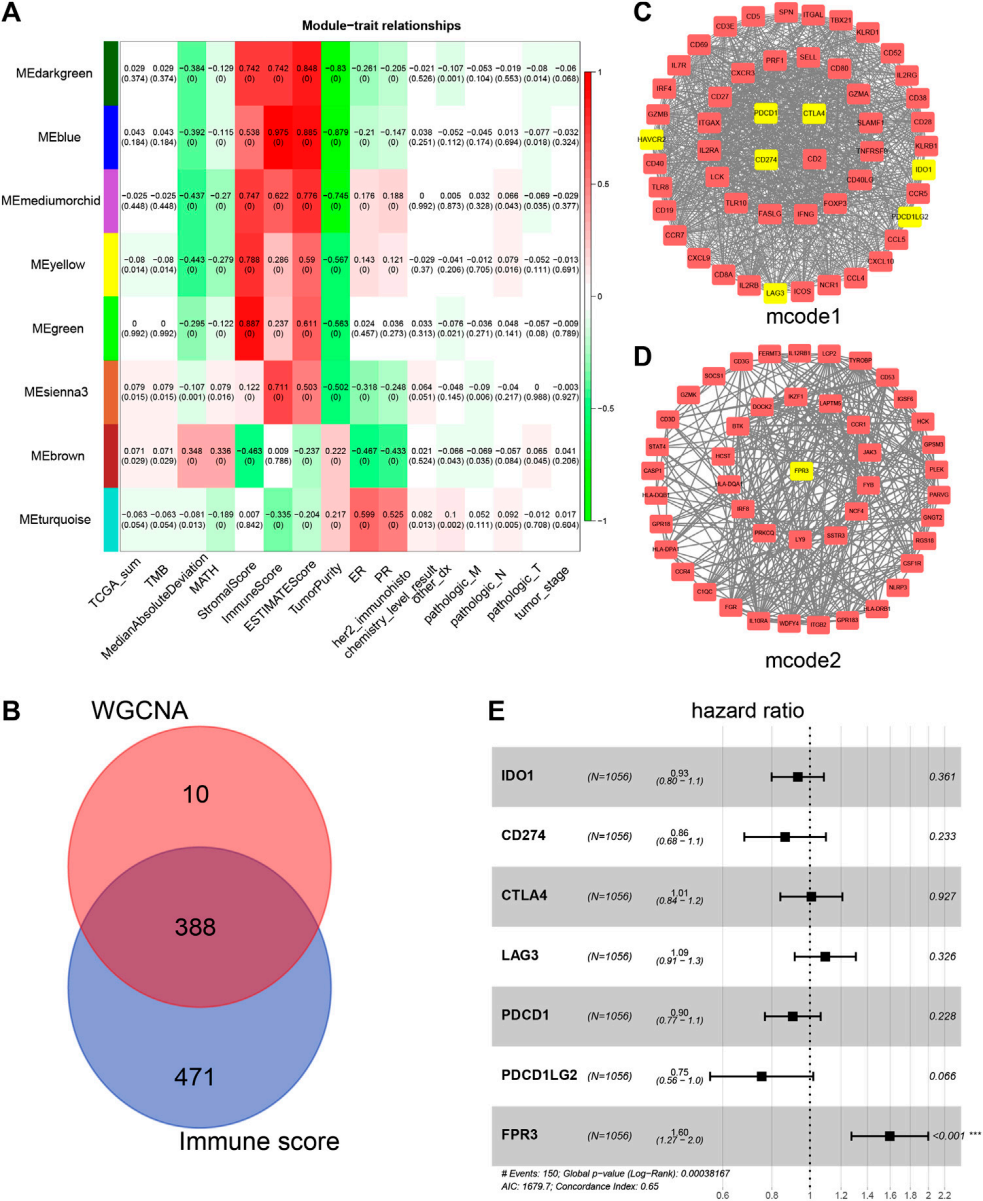
Network visualization plots by WGCNA **(A)** Module-trait associations. Each row corresponds to a gene module, and each column corresponds to a trait. Each cell was labeled by the corresponding correlation coefficient and *p*-value **(B)** Venn diagram shows the genes obtained by the intersection of genes in the WGCNA Meblue module and genes upregulated in high Immune score group **(C–D)** Cytoscape analysis of 388 intersecting genes yielded two most significantly related MCODE modules. Some known immune checkpoints and FPR3 are marked yellow **(E)** Multivariate Cox analysis was utilized to analyze the hazard ratio (HR) of FPR3 and some known immune checkpoints.

Using WGCNA, we sought to identify gene modules that are associated with the Immune score. Among the eight modules, the MEblue was highly correlated with the Immune score (R^2^ = 0.975), Stromal score (R^2^ = 0.538), Estimate score (R^2^ = 0.885), and TMB (R^2^ = 0.043). The MEgreen module showed a higher correlation with the Stromal score (R^2^ = 0.887, Figure 4A). Since the Immune score impacts the survival time of patients (Figure 1G), we identified 388 genes that were overlapped with WGCNA analysis and the high Immune score group (Figure 4B; Supplementary Table S1). To postulate the underlying mechanism for the upregulation of these identified genes, we first used the STRING tool to perform a functional analysis of the PPI network and applied the MCODE plugin in Cytoscape to obtain the two most significant modules. The first MCODE module contains 53 genes, including almost all the established immune checkpoint genes, for example, CD274, PDCD1, CTLA4, and LAG3. (Figure 4C). Intriguingly, many of the immune checkpoint genes predict a better prognosis for breast cancer (Supplementary Figure S3), providing a molecular explanation for the limited effects of targeting immune checkpoints in the treatment of breast cancer.

To identify more attractive prognostic targets in the immune microenvironment, we performed a survival analysis for these 388 genes and found that a total of 101 genes showed a significant difference in patient survival (Supplementary Figure S4). We found that only one particular gene, FPR3, predicts poor prognosis under higher expression, which was included in MCODE module 2 (Figure 4D). To evaluate whether FPR3 is an independent prognostic factor in the immune microenvironment, multivariate Cox analysis was used to analyze the hazard ratio (HR) of FPR3 and some known immune checkpoints (Figure 4E). The six known immune checkpoints do not influence the survival rate of patients (Figure 4E). Only FPR3 falls to the right of the invalid line (hazard ratio >1, *p* <0.001), which means that the higher the FPR3 expression, the worse the patient's prognosis. In addition, the GSE11121 dataset from the GEO database was used to verify the COX analysis. The results also show that FPR3 is an independent prognostic factor (hazard ratio >1, *p* <0.0298), which is more significant than other immune checkpoints (Supplementary Figure S5A).

**FIGURE 5:**
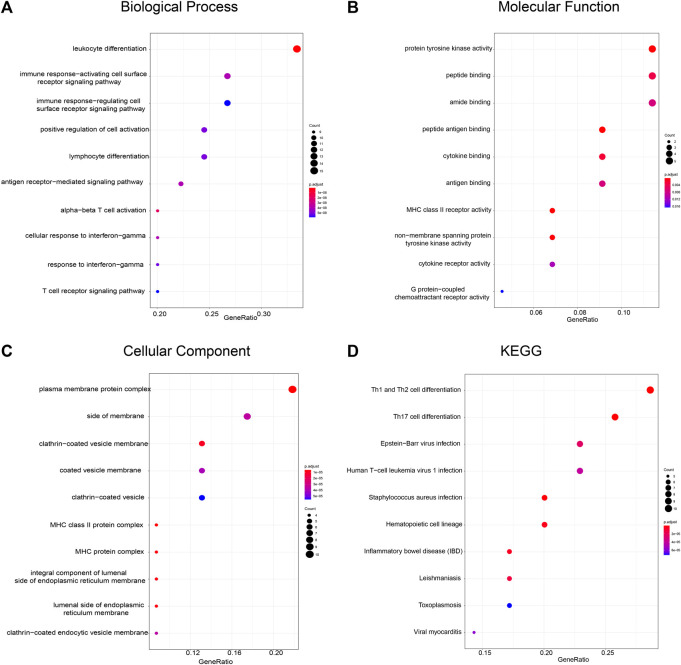
Functional enrichment analysis of FPR3-related gene module **(A)** Biological process **(B)** Molecular function **(C)** Cellular component, and **(D)** KEGG pathways are depicted. The color of the dots demonstrates -Log10(FDR). Red dots indicate smaller FDRs than blue dots. The size of the dots indicates the number of genes enriched in each analysis.

Furthermore, we analyzed the functions of genes in module two using a gene enrichment approach. A total of 46 genes are involved in leukocyte differentiation, immune response-activating cell surface receptor signaling pathway, and positive regulation of cell activation (Figure 5A), with their molecular functions relating to protein tyrosine kinase activity, peptide binding, and amide binding (Figure 5B). These genes are predicted to localize to the plasma membrane (Figure 5C) and are required for Th1 and Th2 cell differentiation, Th17 cell differentiation, and Epstein-Barr virus infection, as indicated by the KEGG pathway analysis (Figure 5D). These results indicate that FPR3, as a component of module 2, may cooperate with other partners in immune-based biological pathways.

### FPR3 Expression in Breast Cancer and the Pathways Enrichment Determined by GSEA

**FIGURE 6:**
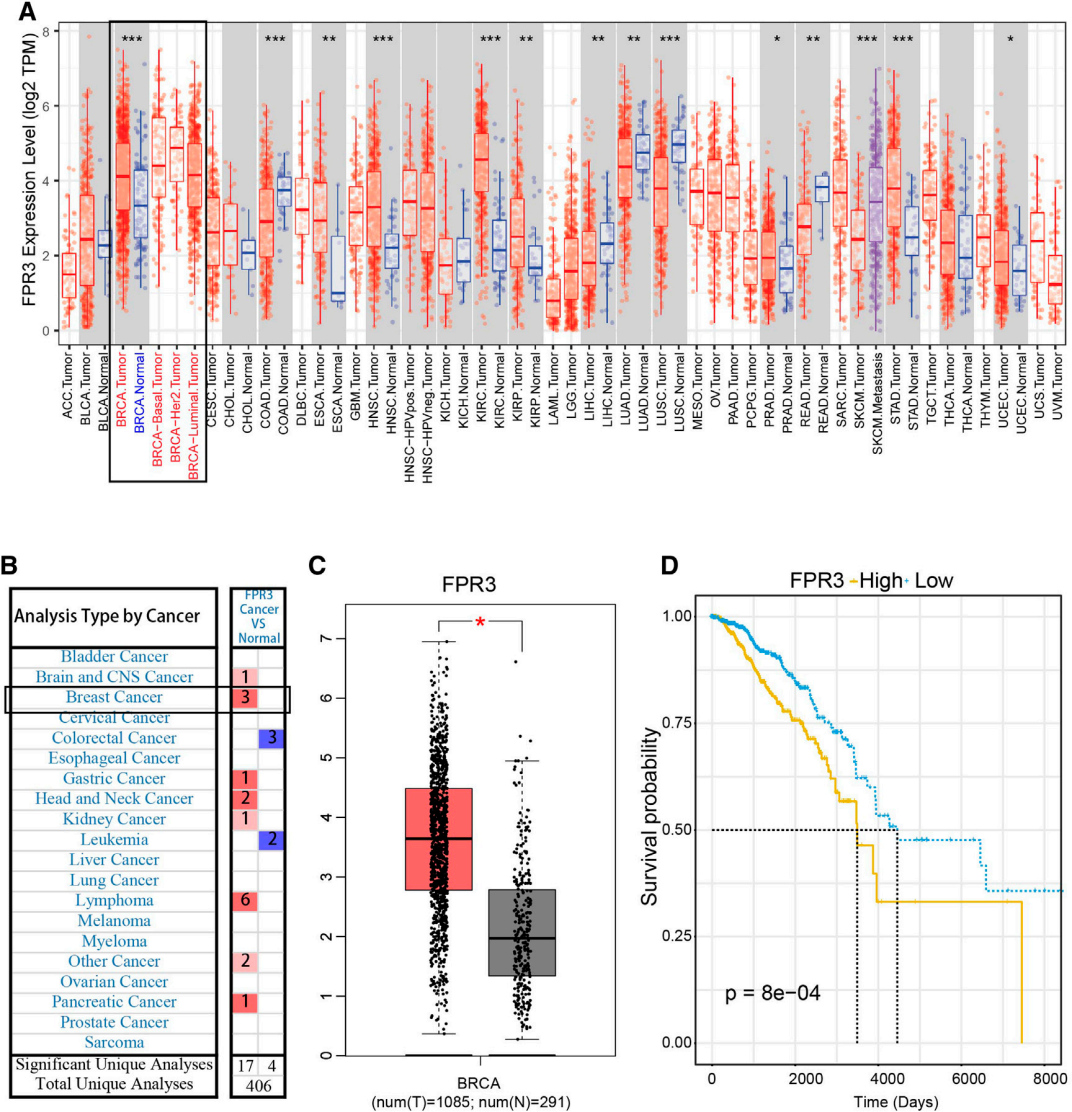
FPR3 expression in breast cancer **(A)** TIMER showed the expression level of FPR3 in pan-cancer, and the expression of FPR3 in total breast cancer cases and different subtypes was higher than that in adjacent normal tissues **(B)** The transcription levels of FPR3 in different types of cancers (Oncomine database). The graph shows the number of data sets in which the mRNA expression of the target gene was significantly up-regulated (red) or down-regulated (blue). The threshold was defined by the following parameters: P < 1E-4 and FC > 2 **(C)** To further examine the expression level of FPR3 between breast cancer and normal tissues, FPR3 was examined using the GEPIA web-based tool (*, *p* < 0.05). Red color indicates tumor tissue and gray color indicates normal tissues **(D)** Kaplan-Meier survival curves were generated for FPR3. The higher the expression of FPR3 (yellow line), the shorter the survival period. *p* value in the log-rank test. OS, overall survival.

We used TIMER to evaluate the expression of FPR3 in pan-cancer. As displayed in Figure 6A, FPR3 is highly expressed in a variety of cancers including different breast cancer subtypes, COAD cancer, and HNSC cancer. As shown in both Oncomine and GEPIA databases, the expression of FPR3 in breast cancer is much higher than that in adjacent normal tissue (Figures 6B,C). The Kaplan-Meier survival curve (Data comes from TCGA) shows that the expression of FPR3 in breast cancer is negatively related to the survival of patients (Figure 6D). Similarly, we also used the GEO dataset to verify the relationship between FPR3 expression and survival. The result is consistent with TCGA data indicating an unfavorable prognosis (Supplementary Figure S5B). The above results suggest that inhibiting FPR3 expression may be useful as an intervention strategy.

**FIGURE 7:**
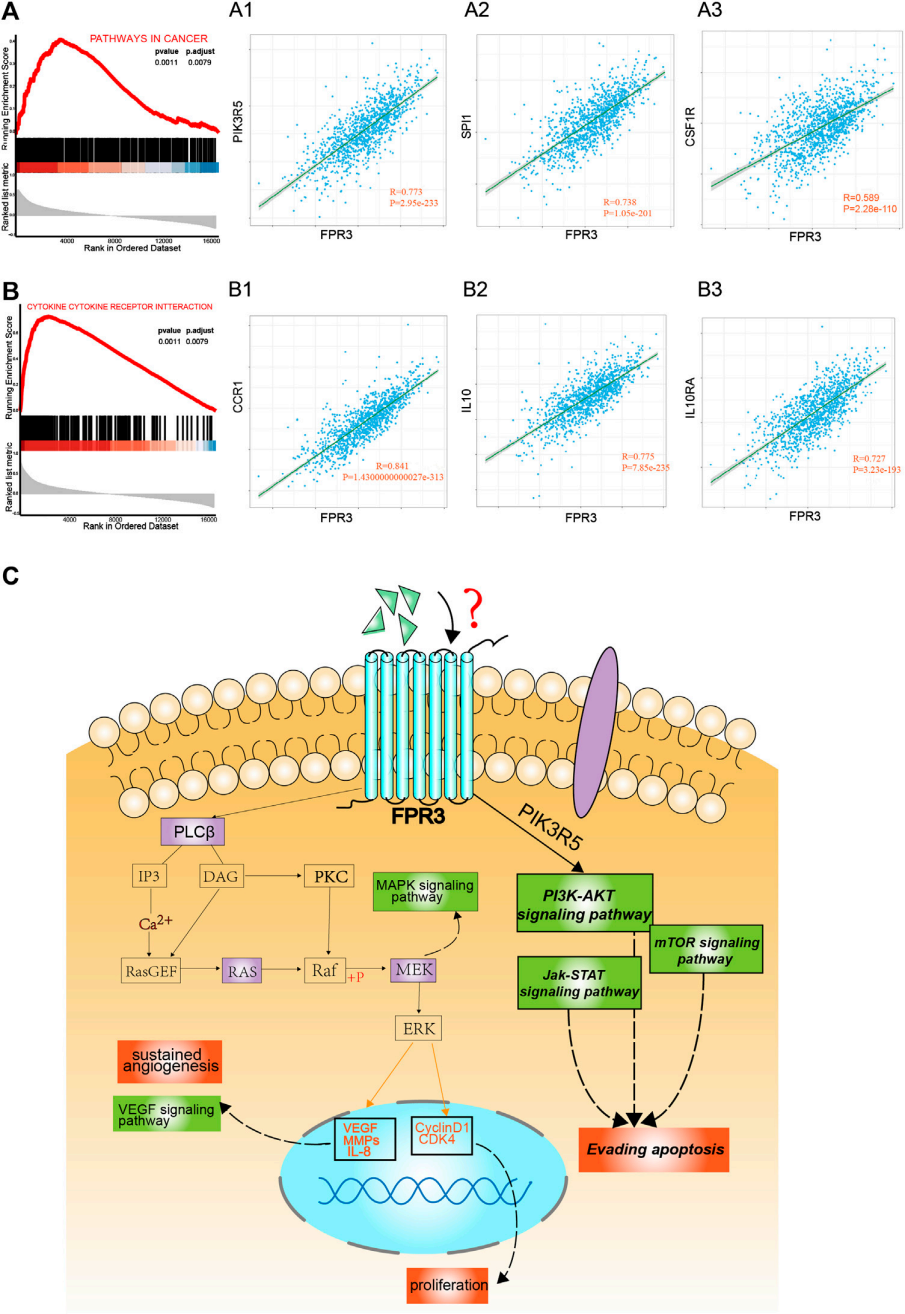
Enrichment plots by gene set enrichment analysis (GSEA). GSEA results showed that higher FPR3 expression was associated with activation of “pathways in cancer” **(A)** (*p* = 0.0011) and “cytokine-cytokine-receptor-interaction” **(B)** (*p* = 0.0011) (A1-A3) shows the co-expression correlation of FPR3 with top genes in the “pathways in cancer”. (B1-B3) shows the co-expression correlation of FPR3 with top genes in the “cytokine-cytokine-receptor-interaction” pathway **(C)** A model diagram was proposed that FPR3 may be involved in the occurrence and progression of cancer *via* G protein-coupled signaling.

To reveal the functional role of FPR3, gene set enrichment analysis (GSEA) was used to analyze the gene expression matrix acquired from TCGA. The samples were divided into high and low expression groups according to the median expression level of FPR3. The top two enriched pathways in the high expression group of FPR3 were “pathways in cancer” (Figure 7A) and “cytokine-cytokine receptor interaction” (Figure 7B), implying that FPR3 upregulation may lead to alterations in cancer-related pathways and cytokine-based immune regulations. We also performed a correlation analysis of FPR3 expression on the GSEA-enriched pathways. In the “pathways in cancer”, PIK3R5, SPI1, and CSF1R are most relevant to FPR3 expression (Figures 7A1–A3). In the “cytokine-cytokine receptor interaction pathway”, the most relevant genes to FPR3 expression are CCR1, IL10, and IL10RA (Figures.7B1–B3). FPR3 may directly or indirectly cooperate with these genes in promoting tumorigenesis. For instance, the high correlation of FPR3 with PIK3R5 implies that FPR3 may promote tumorigenesis through the PIK3R5-mediated G-protein coupled receptor activation (Barberis and Hirsch, 2008). As annotated in the “pathways in cancer”, high FPR3 expression associates with many pathways that promote tumorigenesis and development, such as the VEGF signaling pathway, MAPK signaling pathway, and PI3K-AKT signaling pathway. Because FPRs belong to the classic chemotactic GPCR subfamily, we propose that FPR3 is involved in the G protein receptor coupled pathway to promote carcinogenesis, as shown in the schematic diagram (Figure 7C). Through unknown ligands, FPR3 may regulate breast tumorigenesis through G-protein coupled PI3K or MAPK signaling cascades, participating in a series of carcinogenic processes, such as enhanced proliferation, sustained angiogenesis, and apoptosis evasion.

## Discussion

The tissue microenvironment influences the extent of tumor initiation and development (Quail and Joyce, 2013; Denkert et al., 2018). Four clinically relevant molecular subtypes of breast cancer, luminal A, luminal B, Her2+ type, and basal type, are different in terms of their morbidity, survival rate, prognosis, and tumor biological characteristics. Such patient stratification provides clinical and economic value in breast cancer treatment (Blok et al., 2018). The tumor microenvironment is where the immune system interacts with the tumor (Merlano et al., 2019) and any changes in the composition of the tumor microenvironment may affect the fate of malignant tumors. Therefore, important components of the tumor microenvironment, including stromal- and immune cells, play key roles in cancer progression (Xiong et al., 2018). In the current study, we attempted to identify genes in the tumor microenvironment that are relevant to the survival of breast cancer patients from the TCGA database. Several reports have demonstrated the successful application of estimation algorithms in exploring the gene expression signatures associated with cancers (Jia et al., 2018; Chen et al., 2019; Li and Xu, 2019). We used this algorithm to obtain the Immune score, Stromal score, Estimate score, and tumor purity for breast cancer. We found that these scores significantly correlate with breast cancer subtypes. We used Maftools to establish the correlation between TMB/MATH and breast cancer subtypes. TNM reflects an important aspect of the clinical characteristics of breast cancer. We showed that T stage progression is inversely related to the Immune score, suggesting an important role for microenvironmental factors in tumor progression.

Importantly, we found that overall survival was positively correlated with the Immune score, but not significantly with the Stromal score. This finding suggests that immune factors in the tumor microenvironment play a more important role in determining patient outcomes. WGCNA analysis identified the MEblue module that most relates to the Immune score. Key genes in the MEblue module, including most of all known immune checkpoints, were almost totally upregulated in the high Immune score group. Surprisingly, survival analysis showed that all of these immune checkpoints either had no effect on patient survival or were protective. The FPR3 gene is the only gene that is adverse to the survival of patients. We further found that FPR3 is an independent hazard factor in the immune microenvironment of breast cancer, and its expression is accompanied by the activation of many pathways in cancer, highlighting the essential role of FPR3 in cancer progression.

Immune checkpoint antagonists have altered the way cancer is treated and produced a more durable response, and the FDA has approved a number of them to treat many cancers, but not breast cancer (Lipson et al., 2015). The majority of breast cancer patients respond poorly to checkpoint blockade (Emens, 2018). Moreover, in the clinical evaluation of the safety of PD1 inhibitors, some patients suffer myalgia, fatigue, joint pain, and nausea (Dirix et al., 2018). Although the FDA approved atezolizumab in combination with nab-paclitaxel as a first-line treatment for TNBC in 2019 (Schmid et al., 2020), the efficacy of a single drug was quite poor. Our results also suggest that the expression levels of the known immune checkpoint molecules do not necessarily affect overall patient survival. Therefore, it is of great importance to find novel prognostic molecular markers in the immune microenvironment of breast cancer.

Formyl-peptide receptors (FPRs) belong to the classical GPCR subfamily, and three FPRs have been identified in humans: FPR1-FPR3. Activation of FPR1 and FPR2 by chemotactic agonists elicits a cascade of signaling events that leads to myeloid cell migration, mediator release, increased phagocytosis, and new gene transcription (Cattaneo et al., 2013). FPR1 and FPR2 have been reported to be abnormally expressed in various tumors (Prevete et al., 2015). The expression of FPR1 in gastric cancer tissue is higher than that in normal tissue and is closely related to the survival time of the patient (Yang et al., 2011), and FPR2 is also highly expressed in endometrial cancer and colon cancer (Cocco et al., 2010). However, few studies have been performed on FPR3. FPR3 is expressed in eosinophils, monocytes, macrophages, and dendritic cells, but its function is unclear (Dorward et al., 2015; Nawaz et al., 2020). Several ligands for FPR3 have been identified, including F2L, an acetylated N-terminal fragment of human heme-binding protein (Migeotte et al., 2005), and the neuroprotective peptide humanin (Harada et al., 2004). Interestingly, FPR3 does not interact with formylated chemoattract peptides or ligands for FPR1 or FPR2. Therefore, FPR3 may have a unique functional role (Rabiet et al., 2011). We proposed that FPR3 may regulate the behaviors of tumor cells or immune cells through unspecified ligands in the breast tumor microenvironment. Tumor microenvironment refers to an acidic extracellular fluid system consisting of tumor cells and non-malignant stromal cells (including fibroblast and immune cells) as well as an extracellular matrix, containing a large number of growth factors, peptides, and enzymes. The tumor microenvironment can provide ligands to activate receptors expressed on tumor cells or immune cells. Ligand-receptor binding on tumor cells might activate cancer-related pathways to promote tumorigenesis and development. Thus, activation of FPR3 may regulate various malignant behaviors of cancer cells, including proliferation, avoidance of apoptosis, and sustained angiogenesis, *via* its G-protein-coupled signaling cascades. On the other hand, ligand-receptor binding on immune cells may elicit signaling cascades to cause new gene transcription, mediator release, or cell migration. Therefore, it is necessary to understand the specific properties of the unknown ligand for FPR3, and to make an in-depth analysis of the signaling pathways activated in tumor cells and immune cells. Current work demonstrated that FPR3 is a prognostic marker in breast cancer progression. The detailed mechanisms of FPR3 in breast cancer still require further investigation.

Overall, this study identifies FPR3 as a key immune-related intervention target, improving our understanding of the complex roles of the immune microenvironment in breast cancer progression.

## Data Availability

The datasets presented in this study can be found in online repositories. The names of the repository/repositories and accession number(s) can be found in the article/Supplementary Material.
