# Supplementary material for: Identification of FPR3 as a Unique Biomarker for Targeted Therapy in the Immune Microenvironment of Breast Cancer
Source: Front Pharmacol. 2021 Feb 11;11:593247. doi: 10.3389/fphar.2020.593247 (PMC7928373; doi:10.3389/fphar.2020.593247)
Supplement: Supplementary file 1 [file datasheet1.pdf]

Supplementary Table 1. 388 intersecting genes between the WGCNA Meblue module and genes upregulated in high Immune score group.

Supplementary figure legends

Supplementary Figure 1. Breast cancer cases were divided into two groups based on their average expression of Immune or Stromal scores. Survival curves for DFS (Disease-free survival) (A, B), and PFS (progression-free survival) (C, D) were calculated.

Supplementary Figure 2. Relationships between Immune/Stromal scores and M/N stages. The M and N stages of the tumor are only significantly correlated with TMB, and are not related to Immune score, Stromal score, Estimate score, Tumor purity, and MATH.

Supplementary Figure 3. Survival analysis of six currently known immune checkpoints. The elevated expression of these immune checkpoints in breast cancer generally predicted a better prognosis, which was significant for (PDCD1) but not for (IDO1, CTLA4, LAG3, PDCD1LG2, CD274).

Supplementary Figure 4. 101 genes related to immune score significantly affected overall survival. The most prominent gene, FPR3, was the only predictor of poor prognosis.

Supplementary Figure 5. Survival analysis of FPR3 in GEO dataset (GSE11121).
(A) Multivariate Cox analysis was utilized to analyze the hazard ratio (HR) of FPR3 and some known immune checkpoints. (B) Kaplan-Meier survival curves were generated for FPR3.

**Supplementary Table 1**
CASS4 XCL1 IRF4 TNIP3 CLNK ZAP70 LGALS2 GRAP2 PSTPIP1 SPIB IL12RB1 SLA2 PARVG FASLG NCF4 FGL2 CRTAM ASGR2 CD37 IDO1 PARP15 LILRB2 SIRPB2 GZMM C1QC FCRL6 GZMH BTLA TYROBP GPR65 KBTBD8 PLCB2 C11orf21 CCDC88B MS4A1 EBI3 FGR ADGRE5 C16orf54 ADA2 CD4 DOCK2 IRF1 BANK1 SPN FPR3 CD244 SH2D1A LSP1 IL2RA TRIM22 PLEK RNASE6 NLRP3 CCR1 ARHGAP9 SAMD3 LAX1 CD33 PTPN22 LYN RUNX3 PRKCB HCST PIK3R5 ARHGAP30 GPR183 SLCO2B1 LILRB1 TLR10 CD80 FYB1 RAC2 JAML ADAMDEC1 SAMHD1 LOC107987462 NLRC5 CARD11 LILRB3 FOXP3 SPI1 GZMA MS4A4A CST7 SELL PIK3CG CLEC4E TMEM150B TESPA1 CLECL1 CD38 SERPINB9 TNFRSF9 IKZF1 ZC3H12D TCL1A KCNAB2 LAIR1 EVI2A CD79A GIMAP7 GIMAP1 CXCR3 CTLA4 PLA2G2D HLA-E LILRB4 GIMAP4 IFNG KCNA3 TNFSF13B SELPLG CD28 TRABD2A GBP1 SPOCK2 TBC1D10C C3AR1 SAMSN1 HLA-DPB1 APOBEC3H SASH3 IPCEF1 NCF2 BIN2 CD74 CORO1A CD6 SP140 HLA-DRA EVI2B CTSW TNFSF8 SLC9A9 NCR1 GPSM3 CD52 NCKAP1L RCSD1 LOC105374836 CXCL9 STAT4 CD19 CD40 TNFRSF1B GMFG PIK3AP1 TMEM273 SLAMF1 RASGRP2 GZMB PATL2 FCRL3 CYBB CYTIP SCIMP LAG3 ADGRG5 FERMT3 GPR171 CD53 AKNA CIITA CD247 TRAF1 CXorf21 SOCS1 LPXN CXCL11 C1QB LRMP ARHGAP15 SIGLEC10 P2RY10 HLA-DQB1 SLC15A3 GZMK CD48 LRRC25 MNDA IL18BP GFI1 ITGAX SLAMF7 VSIR TRAT1 MS4A6A APOBEC3C STAP1 INPP5D ITGB7 CD5 CD3G NKG7 BTK CD180 TAGAP IL7R MILR1 HCK MAP4K1 TFEC CXCL10 IL16 TBX21 SLC7A7 LCP2 GAB3 IL10RA RASSF4 KLHL6 ICAM3 CD96 TRPV2 CCL5 PTAFR DPEP2 TTC24 CTSS LCK TIFAB TIGIT HLA-DMA SLA NCR3 HLA-DPA1 HLA-DRB1 PDCD1 CPVL CCR2 TRAF3IP3 IL32 MYO1F NUGGC TMC8 CD300LF PRKCQ CD8B JAK3 LTB CD84 TLR8 LOC105369277 HLA-DOA LYZ HLA-DMB MEI1 LOC107987425 P2RY8 LAPTM5 TMIGD2 CD7 CD27 DOK2 ZBED2 SIRPG S1PR4 RAB33A CD226 MPEG1 CASP1 CD8A APOBEC3G CCR5 SLAMF8 MYO1G STX11 ARHGAP25 GIMAP5 GPR174 CSF2RA B2M LOC102725035 RASAL3 NCF1 CD2 CLEC4A AIM2 ICOS IRF8 IL21R FAM78A PYHIN1 CXCR6 CALHM6 FCRLA HLA-DOB IGSF6 CD200R1 IL4I1 WIPF1 WDFY4 PDCD1LG2 CCL4 WAS FCER1G LY9 LAT2 PRF1 LST1 CMKLR1 CD79B PLAC8 C1QA RIPOR2 IL15RA MFNG SSTR3 FUT7 SRGN ITK EPSTI1 CLIC2 GBP5 CD69 LTA ITGB2 CD72 SIT1 GNGT2 SIGLEC7 CD3D PIK3CD GIMAP6 EOMES VNN2 SNX20 CSF1R TNFRSF8 APBB1IP GPR18 TNFSF14 AOAH HAVCR2 ZBP1 ZNF683 AIF1 TNFAIP8L2 THEMIS2 CD86 RGS18 CD3E IL2RB CD274 POU2F2 TNFAIP3 LY96 SCML4 GBP4 TREML2 CD40LG PDE6G HLA-DQA1 ABI3 NFAM1 KLRD1 SNAI3 C1orf162 ITGAL VAV1 THEMIS PTPN7 IL18RAP CARD16 CSF2RB SLAMF6 PTGER4 CCR7 XCL2 BCL2A1 KLRB1 NLRC3 FGD2 ASB2 PSMB9 HCLS1 UBASH3A SLFN12L ZNF831 CYTH4 BIRC3 FMNL1 ACAP1 PTPRC CCR4 GNLY IL2RG

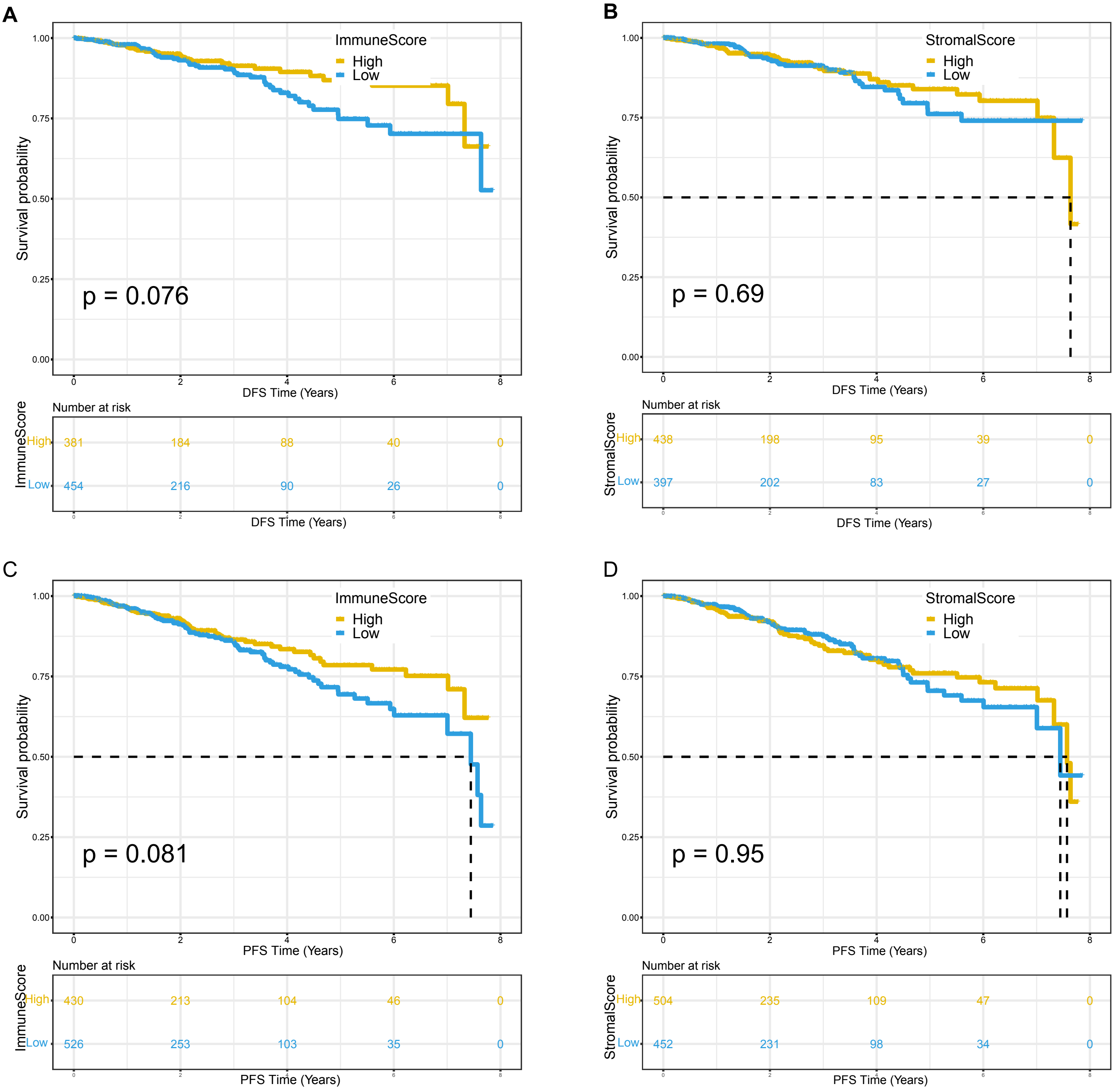

Supplementary Figure 1

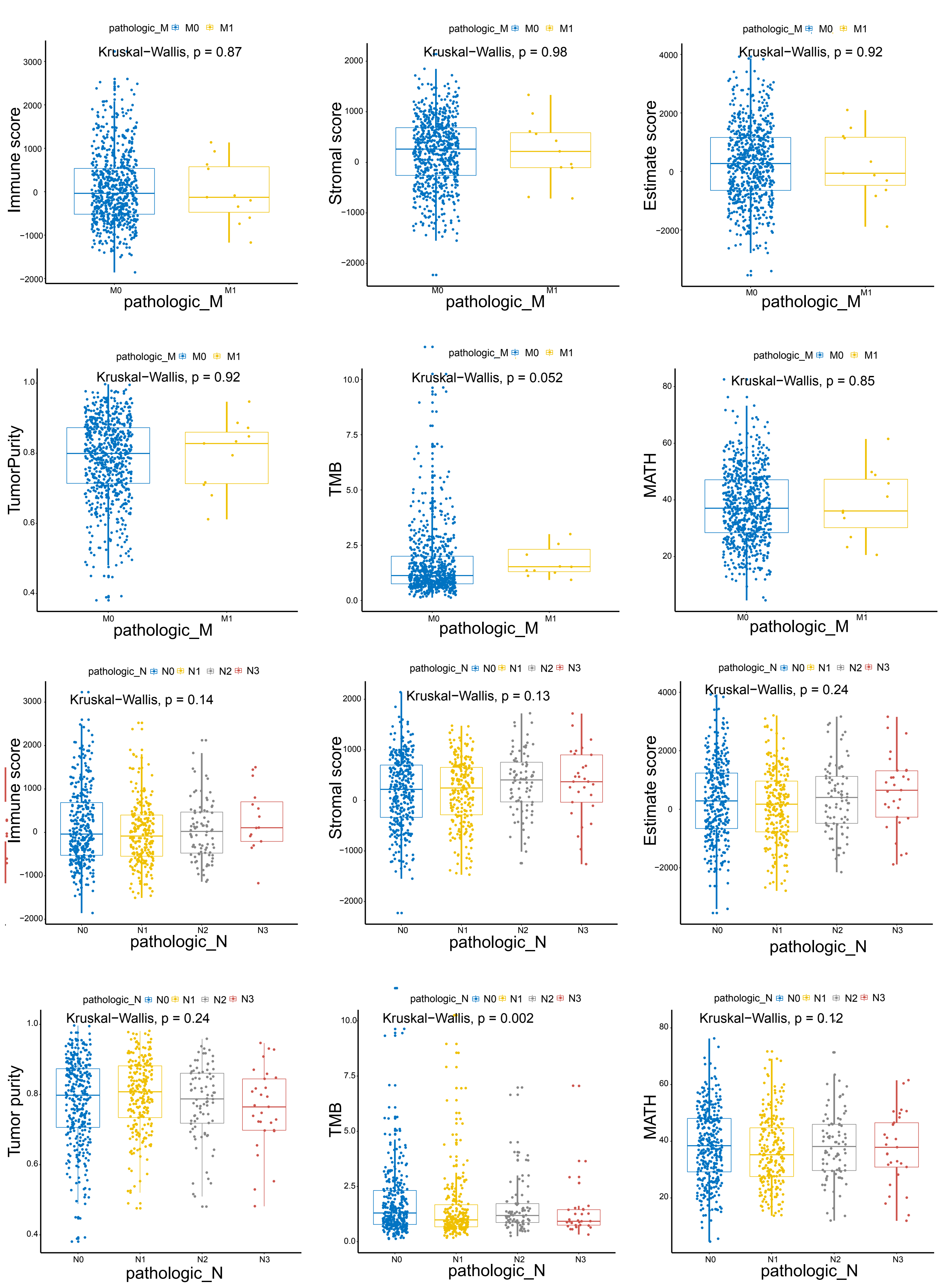

Supplementary Figure 2

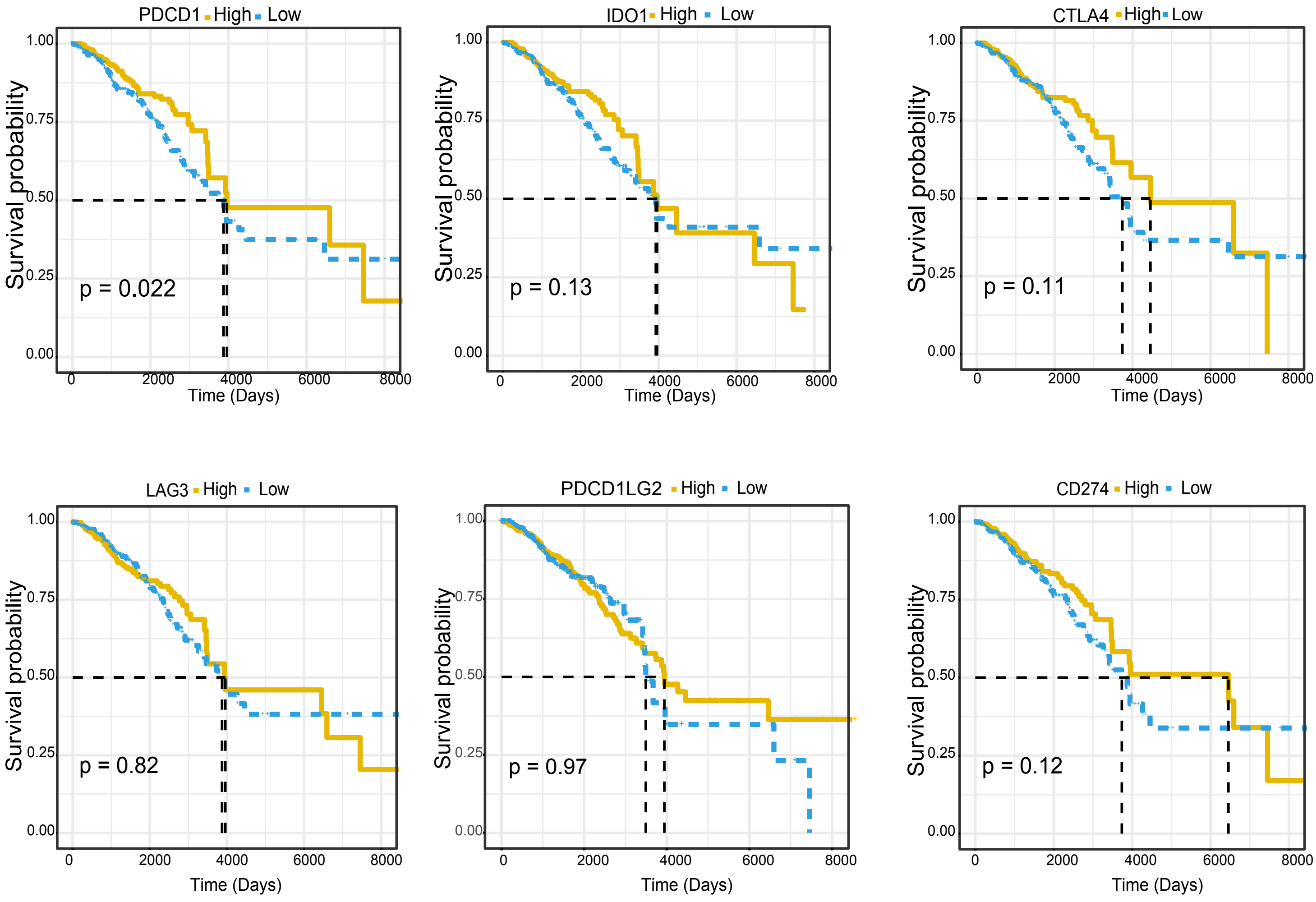

Supplementary Figure 3

gene symbol	p-value
FPR3	0.00080404
CD74	0.000819981
GPR171	0.000974064
CTSW	0.001029724
KLRB1	0.001148958
CD69	0.001271344
CD96	0.002365106
TESPA1	0.003004778
IFNG	0.003852862
STAP1	0.004328895
GRAP2	0.004759999
GZMA	0.005000959
CD247	0.005241784
SELL	0.005349495
PLAC8	0.00555416
CD40LG	0.005733927
GFI1	0.005754999
HCST	0.006286463
CD5	0.00644331
ITGAX	0.006600041
ITGAL	0.00667736
HLA-DRA	0.007431912
UBASH3A	0.007923865
IL7R	0.008540631
TRAT1	0.008873226
SPIB	0.009371092
IL2RB	0.009653969
ARHGAP9	0.009844853
CD2	0.00993288
CLNK	0.010022687
MS4A1	0.010754693
WAS	0.010863213
S1PR4	0.011028502
CD48	0.011218641
MAP4K1	0.011848648
TBC1D10C	0.011983367
HLA-DPB1	0.012206722
ASB2	0.012395351
HLA-DRB1	0.01277977
APOBEC3C	0.012804887
ACAP1	0.012896473
SH2D1A	0.013367721
LY9	0.013471148
STAT4	0.01362768
RAC2	0.013783397
ARHGAP25	0.015508493
AKNA	0.015934968
HLA-DMA	0.0165304
SCML4	0.016827985
TMIGD2	0.01723521
CD3D	0.017710834

gene symbol	p-value
CCR7	0.017819
ZAP70	0.017883
CD79A	0.01863
PARVG	0.01895
SIRPG	0.019464
BTLA	0.020024
CD3E	0.020606
C11orf21	0.020965
CXCL9	0.021301
SPOCK2	0.021494
LTB	0.021553
PDCD1	0.022132
FCRLA	0.022919
APOBEC3H	0.022988
CD226	0.023114
SLFN12L	0.023522
SLA2	0.023642
CD7	0.023873
APOBEC3G	0.024726
C16orf54	0.025638
LOC105369277	0.025638
STX11	0.02631
SIT1	0.026986
MEI1	0.028033
TRAF3IP3	0.028053
SAMD3	0.028164
CD6	0.029179
CXCR6	0.031242
CD19	0.031744
PSTPIP1	0.032661
CD52	0.033497
GZMH	0.034494
TMC8	0.036111
GPR18	0.036888
CYTIP	0.039686
IRF1	0.040198
CD3G	0.040449
TBX21	0.040871
CYTH4	0.041607
LCK	0.042638
SLAMF1	0.042928
FCRL6	0.043063
CD27	0.043184
ITK	0.043668
CD8B	0.044667
PTPN7	0.045604
GZMK	0.045803
BIRC3	0.046925
LGALS2	0.047256
TNFSF14	0.049245

Supplementary Figure 4

A

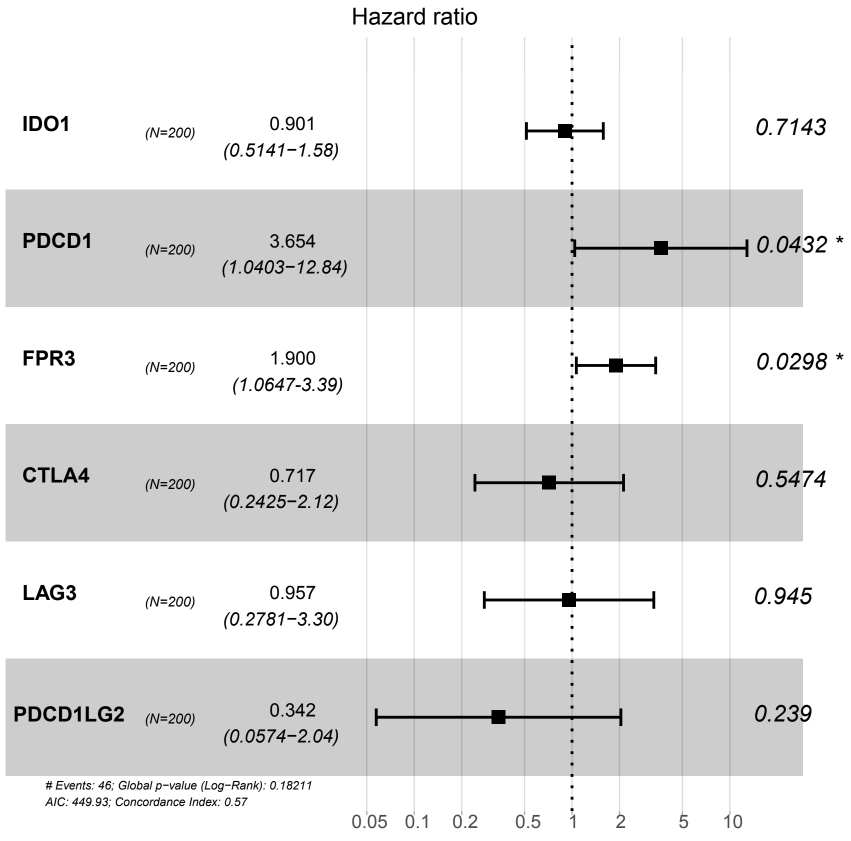

B

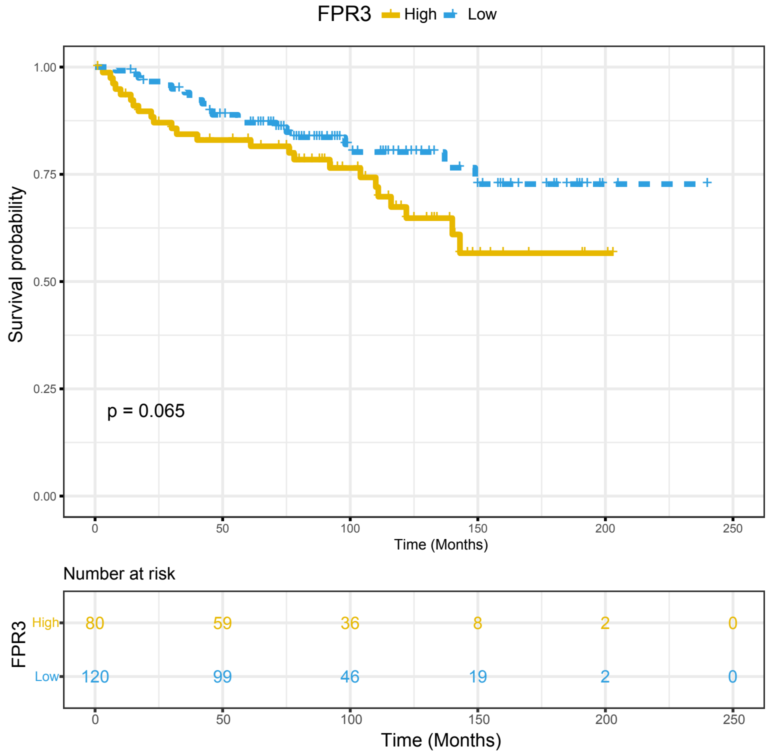

Supplementary Figure 5
